# A Vibration Energy Harvester and Power Management Solution for Battery-Free Operation of Wireless Sensor Nodes

**DOI:** 10.3390/s19173776

**Published:** 2019-08-31

**Authors:** Juan Carlos Rodriguez, Valeria Nico, Jeff Punch

**Affiliations:** 1 Analog Devices, V94 RT99 Limerick, Ireland; 2 CONNECT, Stokes Laboratories, Bernal Institute, University of Limerick, V94 T9PX Limerick, Ireland

**Keywords:** electromagnetic vibration-based energy harvesting, power management, internet of things, preventive maintenance

## Abstract

Electromagnetic Vibration Energy Harvesting (EM-VEH) is an attractive alternative to batteries as a power source for wireless sensor nodes that enable intelligence at the edge of the Internet of Things (IoT). Industrial environments in particular offer an abundance of available kinetic energy, in the form of machinery vibrations that can be converted into electrical power through energy harvesting techniques. These ambient vibrations are generally broadband, and multi-modal harvesting configurations can be exploited to improve the mechanical-to-electrical energy conversion. However, the additional challenge of energy conditioning (AC-to-DC conversion) to make the harvested energy useful brings into question what specific type of performance is to be expected in a real industrial application. This paper reports the operation of two practical IoT sensor nodes, continuously powered by the vibrations of a standard industrial compressor, using a multi-modal EM-VEH device, integrated with customised power management. The results show that the device and the power management circuit provide sufficient energy to receive and transmit data at intervals of less than one minute with an overall efficiency of about 30%. Descriptions of the system, test-bench, and the measured outcomes are presented.

## 1. Introduction

**Figure 1:**
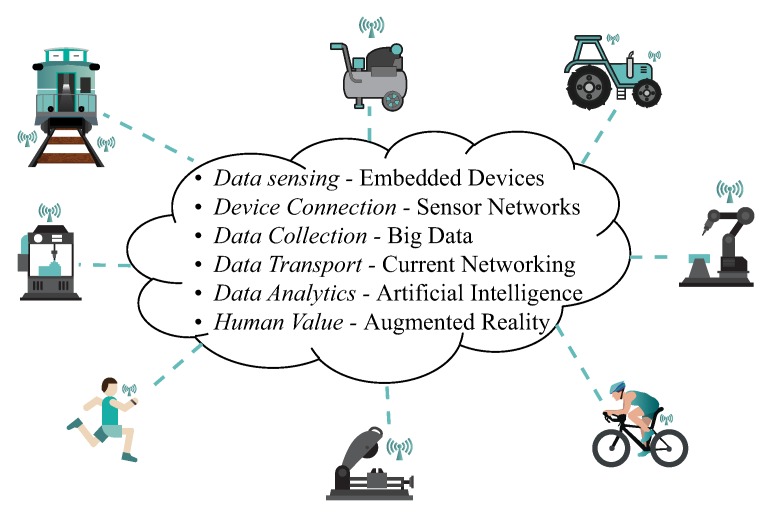
The Internet of Things (IoT).

The Internet of Things (IoT) proposes an environment where individual nodes such as industrial machinery, home appliances, and wearable technology are all interconnected for data collection and exchange, in particular using Wireless Sensor Networks (WSNs) (Figure 1). The current worldwide investment in IoT of around USD 130 billion [1], with up to 7 billion nodes currently connected, has created a thriving market that is forecast to grow to USD 1567 billion by 2025, with 21.5 billion of connected nodes [2]. The IoT market consists of an increasing number of start-up companies, which are defining different stages in the technology development, such as: Sensors, Connection (sensor networks), Storage (solid state), Processing (big-data management), Telecommunications, and Learning (Artificial Intelligence—AI). It has been suggested that the purpose of this evolution is to bring computer systems to a state of *Awareness*. As such, health care, finances, and manufacturing are expected to be re-shaped to include features for Augmented Reality (AR), of which IoT is a foundation.

IoT applications differ according to the place where the data analysis is performed. Thus, there is a differentiation between computation at the edge or in the cloud. Intelligence at the edge of the IoT is often required to increase speed of decisions, add security for data (since it is kept local), and reduce power required for transmission. IoT solutions provided by the integrated-circuit (IC) industry merge different functions (sensing, wireless communications, etc.) in single chips, and are becoming pervasive across the entire ecosystem. These solutions are designed to have low power consumption, and to be highly reliable and “always-on”. Some examples are within IoT products by Texas Instruments (TI) (Dallas, TX, USA) [3], Microchip Technology (MCHP) (Chandler, AZ, USA) [4], and the SmartMesh IP solution range by Analog Devices (ADI) (Norwood, MA, USA) [5].

An underlying need for the resilient interconnection of Sensor Nodes (SNs) at the edge of the IoT is a reliable power supply, and the current standard is the use of batteries. However, expected industrial applications of the IoT such as preventive maintenance require pervasive sensing, with challenges such as a large number of SNs to power, and in places that might be difficult to reach. In such cases, the cost of battery replacement represents a significant barrier to the widespread use of WSNs [6]. Viable alternatives to batteries are renewable power sources, in particular Energy Harvesting (EH) [7]—the collection of small amounts of power (less than 1 W) taken from environmental sources, to feed low power-consumption loads.

Indeed, it is estimated that the operational cost over the product life-time of a harvester-assisted application is reduced to one third of that of a battery-powered one [8]. One of the most popular options is Electromagnetic-Vibration Energy Harvesting (EM-VEH). This technology exploits the relative motion between a coil and a magnet produced by mechanical vibrations that induces AC voltage in the coil. Traditionally these systems have a narrow frequency bandwidth and must be tuned to the dominant frequency of the ambient vibrations—generally in the sub 100 Hz range [9].

To overcome the problem of narrow bandwidth, a two-degree-of-freedom (2DoF) EM-VEH device that exploits multiple masses and a patented velocity-amplification principle has been developed by the authors’ group [10,11,12,13]. In this harvester, harmonic and random excitations can be used as vibration sources for producing higher voltage and power than in prior art.

However, to make this particular technology usable, optimised power conversion (AC-to-DC) and management is needed. Energy conversion schemes for the traditional EM-VEH system assume different conditions than what is true for the harvester in this study. Namely, the equivalent AC voltage level and series impedance of the harvesting source are much larger. This makes the requirements of the power management strategy more stringent, while it is less orthodox and therefore more challenging to be directly solved by an off-the-shelf solution “as it is”. In addition, it is more relevant for the industry of IoT applications to understand the capabilities of the overall EH system when used with real ambient vibration sources, especially those found in industry, to power real WSN loads. These topics have not yet been explored for the particular harvester under discussion.

This paper addresses these needs by introducing a power management technique specific for the multi-modal EM-VEH device reported in [12]. The harvester and its test system are introduced first. Then, the characterisation of the EH system is explored using both ideal input vibrations and vibrations commonly found in industry. The IoT sensor nodes of two different communication technologies, to be continuously powered by the EH solution, are also described. The power management strategy is then developed. Experiments and results are presented before concluding the paper.

## 2. System of Interest

Common vibrational energy harvesters are based on linear mass-spring systems with a narrow frequency response, which requires that they need to be tuned to the main frequency of the external excitation. This usually limits the energy transfer in realistic applications, since ambient vibrations generally have a broad spectrum with several peaks [9]. An alternative to overcome the problem of narrow bandwidth is the two-degree-of-freedom (2DoF) velocity-amplified EM-VEH device introduced in [12,13,14].

**Figure 2:**
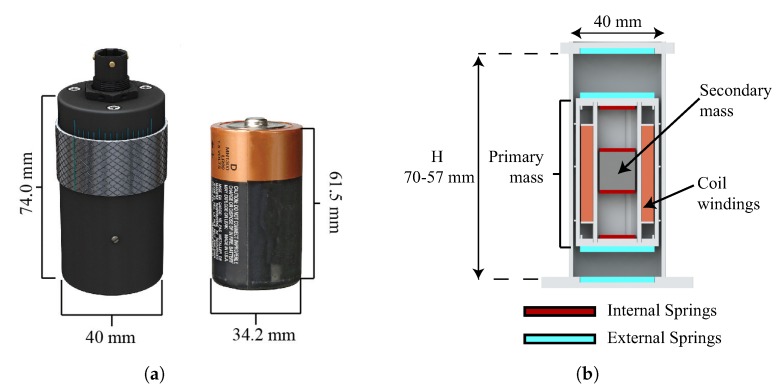
Stokes Power’s VEH-1: (**a**) illustration of the scale of the harvester with reference to a D-battery; (**b**) Schematic of the harvester.

The harvester is currently going through a commercialisation path, and it has been branded by Stokes Power [15], with the commercial name of VEH-1. Figure 2a shows its overall size with reference to a D-battery: diameter 40 mm, height 74 mm.

The device is based on a concept developed by the authors in [12] and its schematic is shown in Figure 2b: it comprises two masses oscillating vertically, one inside the other, between four sets of springs. The distance (*H*) between the external springs (Figure 2b) can be varied and this affects the frequency response of the harvester as shown in Section 3.1. Impacts between the masses can occur, allowing momentum transfer from the heavier mass (the outer mass) to the lighter (the inner mass), leading to velocity-amplification. Electromagnetic transduction is chosen as the conversion mechanism since it is easily implemented in a device that exploits velocity-amplification: the outer mass acts as a housing for the coil and the inner mass is a magnet. According to Faraday’s law, the induced voltage is given by $V_{emf}=Blv$, where *B* is the magnetic field experienced by the coil of total length *l* and *v* is the relative velocity between the coil and the magnet: enhancing the velocity of the inner mass increases the output voltage and the output power ($P_{e}∼v^{2}$).

**Figure 3:**
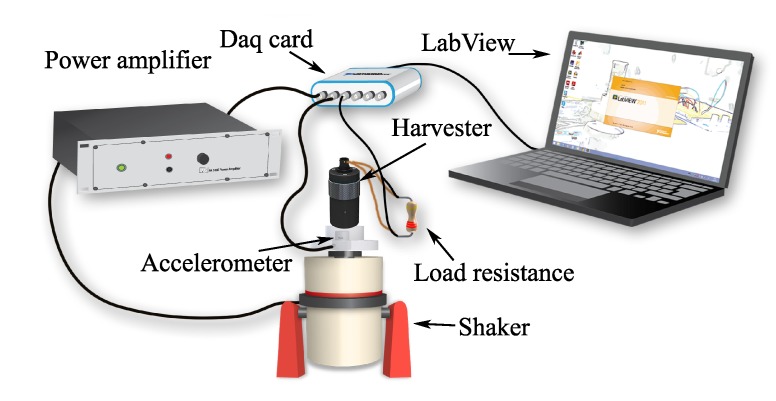
Schematic of the experimental setup.

As shown in Figure 3, the harvester was fixed to a Brüel & Kjaer LDS V406 electrodynamic shaker (Naerum, Denemark) and a PCB Piezotronic accelerometer (Depew, NY, USA), mounted on the head of the shaker below the harvester, was used to provide feedback control to set the acceleration to the desired amplitude level. The output voltage was measured across a variable resistor whose value was fixed to match the optimal load $R_{L}$. Labview (version 2011, National Instrument, Austin, TX, USA) was used to drive the shaker and to acquire signals from both the accelerometer and the harvester.

## 3. Harvester Characterisation

The VEH-1 presented in Section 2 was tested using the experimental setup shown in Figure 3 under sinusoidal excitation over a range of frequencies for different *H* values and under the excitation of an industrial air compressor. For each test, the voltage was acquired across the optimal load resistance $R_{L}$. The root mean square (RMS) output voltage and output power as function of frequency is reported in Section 3.1, while the results obtained under the excitation of the air compressor are reported in Section 3.2.

### 3.1. Harmonic Excitation

The VEH-1 was tested with harmonic excitation at $a_{rms}\;=\;0.4g$ (*g* = 9.81 $m/s^{2}$) for different *H* values that will be referred to as follows:

- Design 1: *H* = 70 mm;
- Design 2: *H* = 60 mm;
- Design 3: *H* = 59 mm;
- Design 4: *H* = 58 mm; and
- Design 5: *H* = 57 mm.

**Figure 4:**
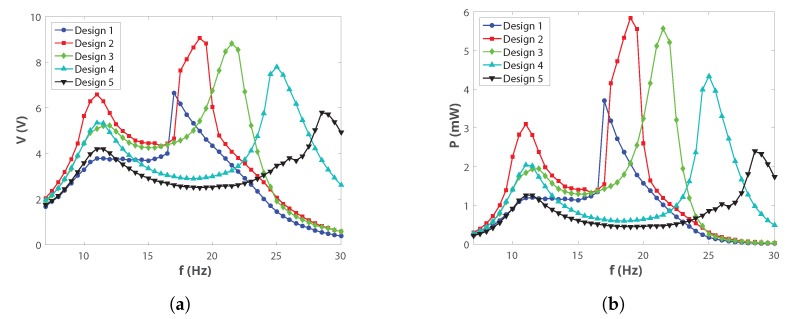
(**a**) RMS voltage and (**b**) power as function of frequency for different *H* values at $a_{rms}\;=\;0.4g$.

Data for *H* values between 70 and 60 mm were not considered since the frequency response of the harvester was not affected in this range. The amplitude of the excitation was chosen because it was comparable with the amplitude of many ambient vibrations [16]. Figure 4 shows the trends of the output voltage and of the output power as function of the five designs for $a_{rms}\;=\;0.4g$.

Two peaks are visible in all the testing configurations, corresponding to the resonant peak of the inner mass (the peak at the lower frequency) and to the resonant peak of the outer mass (the peak at the higher frequency). The peak at the lower frequency is not affected by a change in *H*, as expected, since the size of the cavity inside the outer mass was not varied. The peak at the higher frequency, instead, shifts to the right when the distance between the external springs is changed: reducing *H* had the same effect as increasing the stiffness of the springs. The harvester can hence be tuned to the dominant frequencies of ambient vibrations by varying a geometrical parameter: for example, the second resonant peak shifted from 19 Hz in Design 2 to 29 Hz in Design 5, as shown in Figure 4.

In the next subsection, the vibrations of an industrial compressor are reproduced using the electrodynamic shaker to show that the device could generate enough energy to power wireless sensor nodes.

### 3.2. Industrial Air-Compressor Vibrations

**Figure 5:**
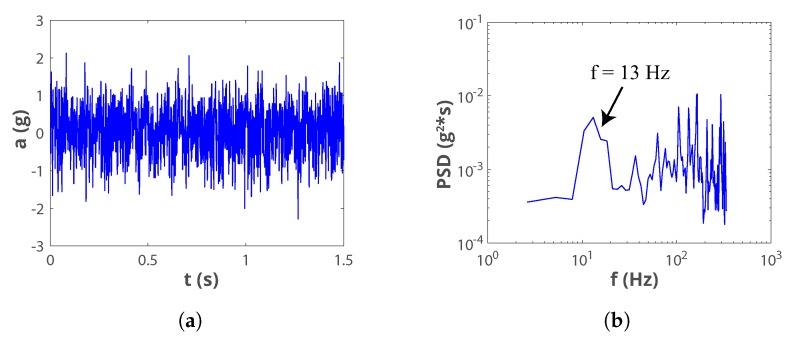
Vibrations measured on an industrial air compressor: (**a**) Time series; (**b**) Power spectral density.

Typical sources of mechanical vibrations in industry are devices linked to motors. While industries like mining and concrete-block manufacturing have machinery with stronger vibrations, the most commonly found equipment are pumps, fans and compressors. The acceleration pattern of the vibrations on a medium-range-power air compressor was measured in situ, in a real industrial environment; and it is shown in Figure 5a. The RMS of the acceleration is $a_{rms}$ = 0.73*g* and the dominant frequency is 13 Hz as visible from the Power Spectral Density (PSD) shown in Figure 5b.

**Figure 6:**
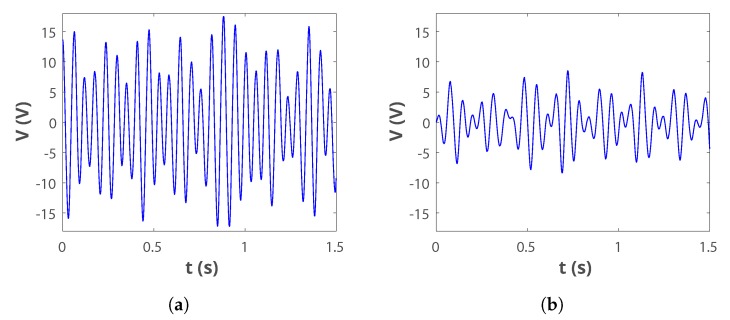
Output voltage delivered by the harvester: (**a**) Open circuit configuration; (**b**) Measured across $R_{L}$ = 12,050 Ω.

The measured acceleration pattern was then used to emulate these mechanical vibrations in the laboratory, using the experimental setup shown in Figure 3. Design 1 was used to test the VEH-1 because the resonant frequency in this configuration was close to the main peak of the ambient vibrations. The open circuit voltage generated by the harvester, shown in Figure 6a, has RMS/peak voltage levels of 8.4 V/17 V. Figure 6b additionally reports the voltage measured across a load resistance $R_{L}$ = 12,050 Ω which has approximate RMS/peak voltage levels of 3.45 V/8.6 V. The harvested power, calculated using $P=V_{rms}^{2}/R_{L}$, is 0.98 mW. In this study, this level refers to the absolute maximum power that the harvester is able to deliver potentially, before any power conditioning.

## 4. Output Low-Power Loads

Interconnection of sensor nodes at the edge of the IoT is implemented using different wireless communications protocols. These wireless networks, based on their range, are classified as short-range or long-range. While long-range technologies like LoRA and Sigfox have demanding requirements (as they are preferred for critical applications), short-range solutions like BLE and WiFi have additional challenges like harsh industrial environments in private or non-proprietary bands [17]. Both paradigms are represented in the IC industry, with a broad and flexible range of products for wireless connectivity. Some examples are the LTC5800-IPM SmartMesh IP System-On-Chip (SoC) by ADI [18] (to build on the 802.15.4e standard), and the sub-1 GHz transceiver (Sigfox enabler) by ST Microelectronics (ST) (Geneva, Switzerland) [19]. Two other representative communication technologies have been chosen as the low-power loads in this study, and are presented next.

### 4.1. LoRaWAN End-Device Node

The first load is a LoRaWAN end-device developed by Pervasive Nation, an Irish IoT testbed operated by the CONNECT Research Centre, for use in its long-range national network through a custom gateway. This small form-factor interface board reports data from a selection of on-board general purpose sensors (accelerometer, Hall sensor, temperature and humidity) over LoRaWAN [20].

**Figure 7:**
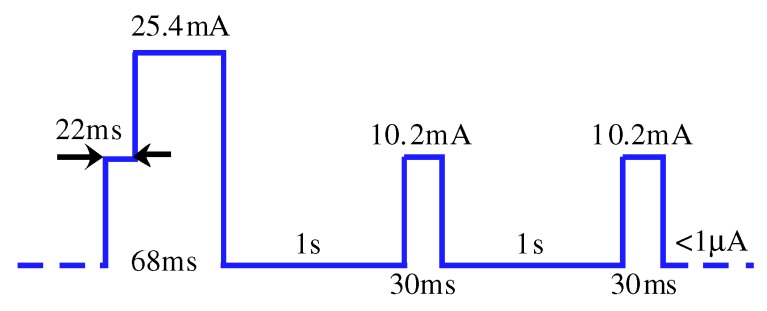
LoRa board current consumption profile in one *Rx/Tx* cycle.

The LoRa board feeds off a 3.3 V (nominal) DC voltage, in the range 2.5 to 3.6 V, which is originally provided by a coin-type battery. The operation mode within the protocol was modified so that the board implemented minimum power consumption with a single sensor (magnetometer). The practical current consumption profile of the board, for one round of data Reception/Transmission (*Rx*/*Tx*), was measured, and it is depicted in Figure 7.

The device goes through this cycle and reverts to its sleep mode, where current consumption decreases to a few micro-Amps (negligible). Table 1 identifies the average power consumed by the board for different sleep times (at a nominal input voltage of 3.3 VDC). Usual *Rx*/*Tx* rates in LoRaWAN applications admit sleep times of up to 15 min [21].

### 4.2. Bluetooth Sensor Node

The second load is TI’s CC2650 SensorTag. This is a more standard IoT device that supports 10 low-power sensors, embeds expansions for customised IoT applications, and it typically feeds off a coin-type battery with a 3 V (nominal) DC voltage, in the range 2 to 3.4 VDC. The board is able to connect with a mobile app via Bluetooth, in a short-range network where the transmission rate and number of sensors enabled in the session can be modified [22].

**Figure 8:**
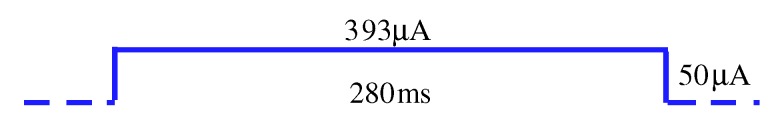
Bluetooth board current consumption profile in one *Rx*/*Tx* cycle.

Using the board features, an application with three simultaneous enabled sensors (ambient light, and local and ambient temperatures) was configured. The current consumption profile, for one round of data *Rx*/*Tx* is presented in Figure 8 (for a nominal 3 VDC input voltage), and the power consumption for different sleep times is presented in Table 2.

## 5. Power Management for EM-VEH

The SNs targeted by the proposed EM-VEH technique have embedded electronics designed to be powered from a coin-type battery of ∼3.3 VDC. Since the output voltage of the VEH-1 is of AC nature, the power management (PM) strategy must first implement AC-to-DC electrical energy conversion, and then further DC voltage regulation to deliver a reliable DC supply near 3 VDC, while maximising the energy transfer.

To solve this problem, the IC industry offers a wide range of Power Management Integrated Circuits (PMICs) specifically for EH. These devices integrate ultra-low power conversion schemes with control techniques that are customised for EH applications. This is because an SN can operate continuously only if the energy production is equal to or superior to the consumption, which is challenging for any EH supply and hence low-power-management strategies are required. On the load side, this is achieved by implementing customised scheduling with data *Tx*/*Rx* implemented only every several seconds/minutes (Section 4) [23]. On the supply side, Maximum Power Point Tracking (MPPT) techniques, usually implemented by the PMIC, assist with the power delivery.

**Figure 9:**
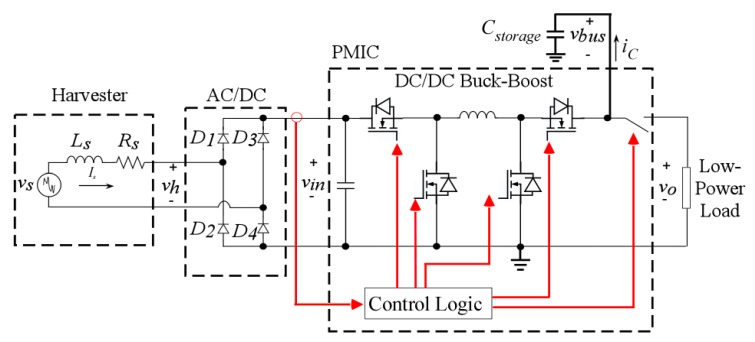
Schematic of the power conversion and management strategy.

The majority of EH PMICs available in the market are specifically designed for solar photovoltaic (PV), thermoelectric (TE), and piezoelectric vibrational energy harvesting (PE-VEH), with very few options specifically for EM-VEH. The input electrical conditions for which such commercial PMICs are designed are quite different from the ones for the harvester in this study. PV and TE harvesters have very low DC input voltages with relatively higher input currents, while PE-VEH features high AC voltages with lower input impedances. In contrast, the VEH-1 features a much higher AC input voltage (Figure 6) and input impedance. The latter is due to the high number of turns in the coil of the VEH-1 design, which makes the coil resistance ($R_{s}$) and inductance ($L_{s}$) in the typical dynamic electrical model of an EM-VEH harvester (shown in Figure 9) high. In this study, they were measured to be 3840 Ω/282 mH. This is larger than the usual few ohms/milli-henries in typical EM-VEH designs [24]. This fact makes the implementation of an MPPT strategy vital and particularly difficult, since the high input impedance yields a more unstable input DC voltage.

Another challenge is that even the few PMICs in the market that are suited for EM-VEH do not implement an adequate MPPT strategy. Theoretically, MPPT involves matching the impedance of the load observed by the harvester to that of the harvester. Commercially, this is typically achieved by well-known algorithms such as perturb-and-observe, which adjusts the input voltage until the measured input power is maximised. MPPT was originally proposed for PV energy transfer [25], and has not been deeply explored for EM-VEH in general.

To overcome these challenges, the PM in this study has been implemented by adapting commercially available solutions to the special conditions of the VEH-1 harvester. A commercial survey revealed the most suitable commercial PMICs for this EM-VEH application to be the BQ25570 by TI [26], the LT3331 by ADI [27], and the SPV1050 by ST [28]. A schematic of the PM strategy that was finally used in this work is introduced in Figure 9, and the basic features of the system are detailed next.

The first stage in the PM is front-end AC-to-DC energy conversion. Several PM methods have been proposed in the literature on EM-VEH; from which the synchronous active rectifier is one of the most efficient strategies, while also providing further regulation [29]. However, this technology has not matured enough yet to be found in commercial PMICs. Another form of active rectification is the Negative Voltage Converter (NVC)—a bridge rectifier implemented with MOSFETs (and an active diode) that reduces the device voltage drop, when compared against using a diode rectifier [30].

These solutions are based on the idea that EM-VEH produces AC voltages of peak values less than 1 V [9], which is true for traditional harvesters of this type. However, the VEH-1 is able to deliver higher voltages (open circuit peak voltage $V_{peak}$ = 17 V, Figure 6a) in this particular application. Experimental work confirmed that the use of an NVC presented no particular advantage when compared to a (Schottky) Diode Bridge Rectifier (DBR), which was finally chosen as seen in Figure 9. The rectified version of the harvester voltage $v_{h}$ in Figure 6a, measured across a filter capacitor, is $v_{in}$ and it has an average value of 15 VDC. This average open-circuit value is less than $V_{peak}$ = 17 V (Figure 6a) because the input signal is not perfectly sinusoidal. In addition, the value of $v_{in}$ is further reduced due to the forward voltage drop caused by the rectifiers (of <240 mV per diode due to the very low input current).

The second stage in the PM implements DC-to-DC voltage regulation. Again, due to the very low voltage outputs of traditional EM-VEH devices, the most common topology for DC-to-DC conversion in several commercial PMICs is a boost converter [31], usually rated for 5 V, which is not adequate for the voltage range of the VEH-1 (∼17 V in Section 3.2). Instead, the PM technique in Figure 9 implements DC-to-DC buck-boost conversion (allowing input voltages up to 20 VDC). This conversion circuit has to be controlled with an adequate MPPT strategy.

The proposed PM technique in Figure 9 realises an MPPT from the harvesting source that stores the energy in a super-capacitor $C_{storage}$, and delivers regulated 3.7 VDC to the low-power load. This output voltage was chosen following the maximum level admissible in the range of the loads (Section 4). As soon as the harvester source is connected, the system goes through several intervals that will be presented next to complement the understanding of the PM strategy.

### 5.1. Cold-Start Interval

**Figure 10:**
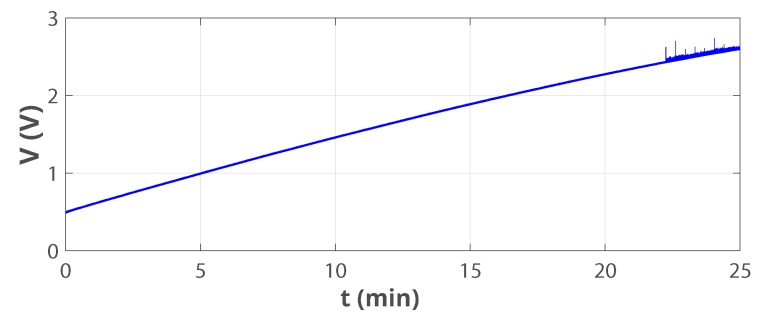
Bus voltage during cold-start.

The (rectified) input from the harvester $v_{in}$ is directly connected to the storage device $C_{store}$ so that $v_{in}=v_{bus}$ during the cold-start interval $\Delta t_{cs}$, which runs for: $V_{in,min}<v_{bus}<V_{EOC}$. $V_{in,min}$ and $V_{EOC}$ are, respectively, the minimum input rectified voltage, and an end-of-charge voltage level which is defined by the PMIC in use. In this study: $V_{in,min}$ = 150 mV and $V_{EOC}$ = 2.6 V. Figure 10 shows the bus voltage on the storage super-capacitor during cold-start. It took around 22 min for $C_{storage}$ to charge to $V_{EOC}$ under the excitation detailed in Figure 5.

### 5.2. Switching Interval

During the switching interval $\Delta t_{sw}$ after the bus voltage has reached $V_{EOC}$, the internal buck-boost DC-to-DC converter of the PMIC operates according to the MPPT algorithm: the converter will stop switchinxg for a period $T_{sample}$ every certain time $T_{tracking}$. In this study $T_{sample}$ = 400 ms and $T_{tracking}$ = 16 s.

During $T_{sample}$, $v_{in}$ is allowed to reach its open-circuit voltage $V_{oc}=$ 15 V (maximum harvester voltage), in order to record this level. Once this interval is elapsed, the converter operates again, setting its own impedance such that $v_{in}$ stays as close as possible to the (previously) programmed maximum power point voltage $V_{MPP}$, which is a percentage ($MPP_{ratio}$) of $V_{oc}$, such that:

(1) $$V_{MPP}=MPP_{ratio}\times V_{oc}.$$

The ideal $MPP_{ratio}$ for EH using PV panels and Thermoelectric Generators (TEGs) is well identified in the literature to be around 50% and 80% of $V_{oc}$, respectively [23]. For EM-VEH, however, there is no general agreement.

A particular analytical description of the ideal $MPP_{ratio}$ for the VEH-1 can be developed by recognising that, in the dynamical electrical model of the harvester (as in any other electrical system), the inductive impedance of the coil $L_{s}$ is negligible if it is at least 10 times smaller than its resistive impedance $R_{s}$ i.e., $R_{s}>10\times \omega_{o}L_{s}$, for a single-mode excitation of frequency $f_{o}=\omega_{o}/2\pi$. In the VEH-1, $L_{s}$ is negligible for excitation frequencies smaller than 611.15 Hz, which is between the expected range for the experiments in this paper (in Figure 5), and so the equivalent series impedance of the harvester block in Figure 9 is simply $R_{s}$.

**Figure 11:**
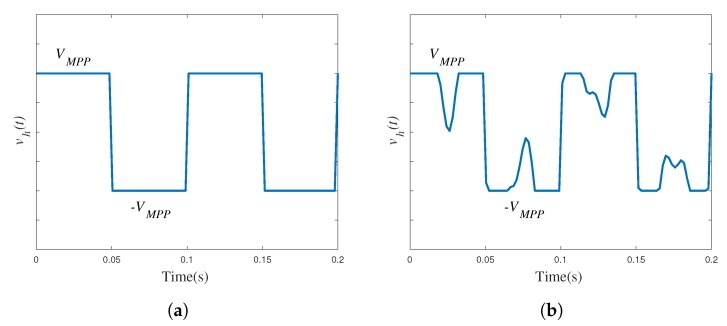
Reflection of the PMIC input voltage into the harvester AC terminals: (**a**) Single-frequency excitation; (**b**) Multiple-frequency excitation.

If a resistive load $R_{L}$ is connected across the harvester, maximum power occurs when the impedance of the load matches that of the input source ($R_{s}=R_{L}$), so the conditions for maximum power transfer when this harvester is single-frequency excited are:

(2) $$\begin{array}{cc} R_{L} & =R_{s}, \end{array}$$

(3) $$\begin{array}{cc} V_{h}\left(peak\right) & =V_{s}\left(peak\right)/2, \end{array}$$

where $V_{h}\left(peak\right)$ is the peak voltage across the load observed by the harvester. This load is determined by the MPPT operation of the PMIC in the circuit of Figure 9 by which effectively $v_{in}$ is kept at a constant value $V_{MPP}$, and the shape of $v_{h}\left(t\right)$ (the voltage reflected into the AC-side of the DBR) is as in Figure 11a.

This shape is close to a square waveform, which, according to its Fourier analysis, has a fundamental amplitude of:

(4) $$V_{h,1}\left(peak\right)\approx \left(4/\pi \right)V_{MPP}.$$

Approximately, the peak voltage in Equation (4) is observed by the harvester. Therefore, for a single-mode excitation, the MPPT conditions in Equation (3) dictate that under MPPT: $\left(4/\pi \right)V_{MPP}\;=\;V_{h}\left(peak\right)\approx V_{s}\left(peak\right)/2$, and so:

(5) $$V_{MPP}\approx 0.39V_{s}\left(peak\right).$$

This analysis shows that, if the harvester is single-frequency excited, the ideal $MPP_{ratio}$ is around 39%.

In reality, the VEH-1 cannot be represented by a single-frequency sinusoid, but instead it is a composition of different *h* harmonics: $v_{s}\left(t\right)\;=\;\sum_{h}v_{s,h}\left(t\right)$. For this input, it can be proved that the power in a resistive load $R_{L}$ (again, considering $L_{s}$ negligible) is $P_{L}\;=\;\left(R_{L}/\left(R_{s}+R_{L}\right)\right)\sum_{h}V_{s,h}^{2}\left(rms\right)$. It follows that the conditions for MPPT in Equations (2) and (3) still apply. However, in multi-modal excitation, the shape of $v_{h}\left(t\right)$ cannot be expected to be perfectly square anymore. An example of the shape of a multi-frequency, rectified waveform observed experimentally is presented in Figure 11b. For that shape, the peak of the fundamental sinusoid observed at the input of the PMIC is less than $\left(4/\pi \right)V_{MPP}$ (since the Fourier coefficients are calculated by integral analysis). Consequently, $V_{MPP}$ will have to be slightly higher than in Equation (5) and it can be said with some level of certainty that, for the multi-frequency VEH-1 harvester, the ideal $MPP_{ratio}$ is expected to be larger than 39%. How large it will be will depend on how distorted the input voltage $v_{in}$ is with respect to a square signal.

**Figure 12:**
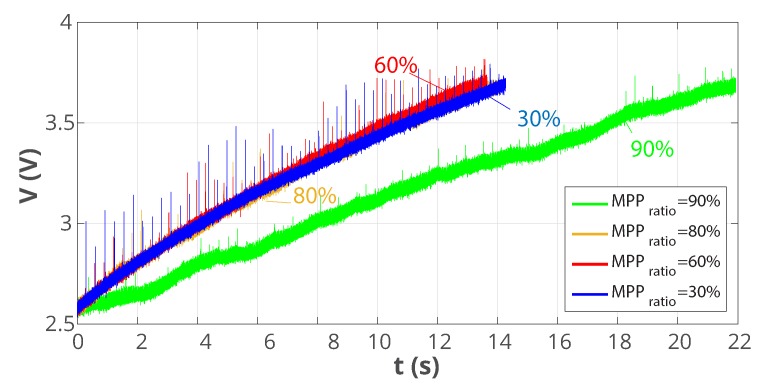
Bus voltage during switching interval for different MPPT ratios.

This ratio was ultimately identified experimentally. Figure 12 shows the bus voltage during the switching interval, for different MPP ratios, in the real experiment. The results show that the optimal $MPP_{ratio}$ is around 60%, so this is the value to be programmed in the PMIC.

### 5.3. Operational Interval

Once $v_{bus}$ across the storage device rises above $V_{EOC}$ (around 3.6 V as defined in Section 4), an internal output switch of the PMIC closes, making $v_{bus}=v_{o}$, so the energy in $C_{storage}$ is finally transferred to the load, and thus starting the operational interval $\Delta t_{op}$.

During this interval, the DC-to-DC converter is enabled/disabled intermittently such that $v_{bus}=v_{o}$ remains close to 3.6 VDC based on internally-defined hysteresis levels. Furthermore, an undervoltage protection opens the output switch when $v_{bus}=v_{o}$ falls below a lower threshold level of 2 V, hence returning to either cold-start or switching modes, depending on the voltage drop. The value of $C_{storage}$= 0.1 F was designed considering this behaviour.

The response of this particular interval is unique for each one of the loads described in Section 4, and the results are reported in the next section.

## 6. Experimental Results

**Figure 13:**
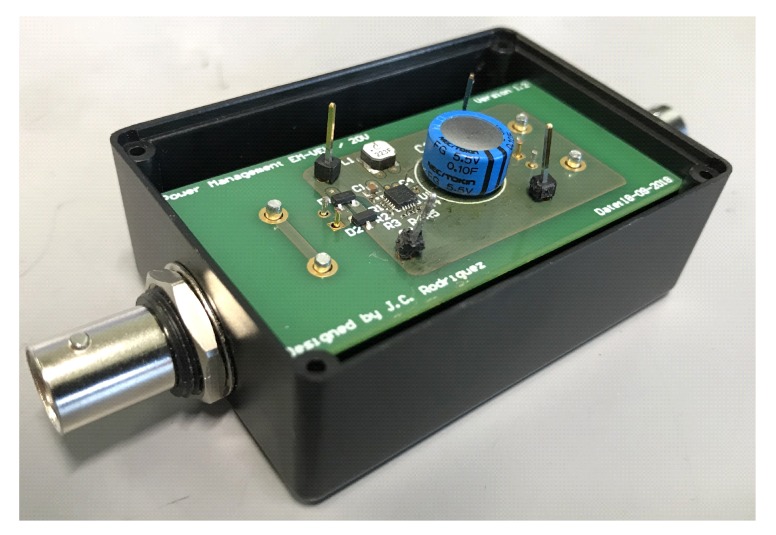
Power management PCB.

The power management solution proposed for the VEH-1 in the previous section was implemented in a 60 mm × 30 mm PCB, enclosed in a 29 mm × 74 mm × 49 mm box with two I/O BNC connectors, as seen in Figure 13. In the following subsections, the distance between the node and the receiver was two meters.

### 6.1. LoRaWAN Node

**Figure 14:**
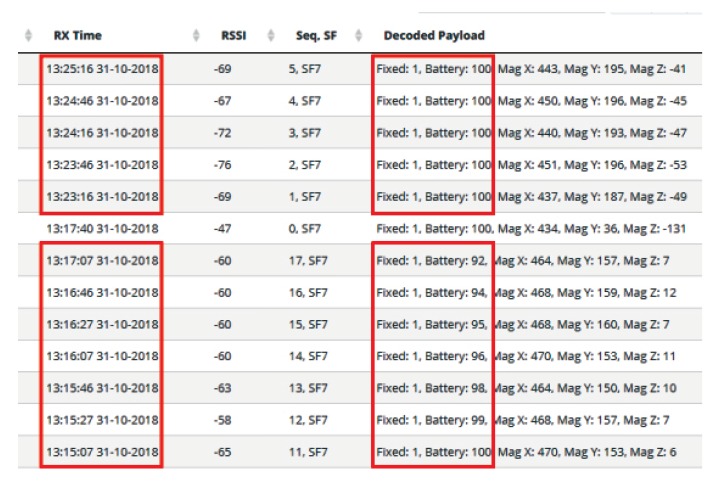
LoRaWAN broadcasting results.

During the operational interval, the LoRa board transmits the data of its magnetometer sensor to a gateway which makes the data available over the internet through the LoRaWAN protocol. Different data rates (sleep times) were tested on the board to find the minimum for which the board was kept energised. The screen shot of the data in Figure 14 shows how, after changing from a sleep time of 20 to 30 s, the embedded “battery charge“ measurement switches from a dropping charge (100% to 92%) to a stable 100% charge. Hence, 30 s is the minimum duty cycle achievable in this application. Note that this is far more frequent than the usual 15-min data rate in the protocol.

### 6.2. Bluetooth Node

**Figure 15:**
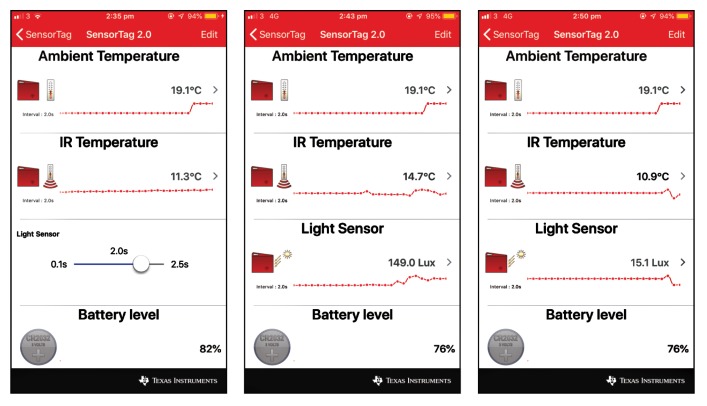
Broadcasting results with the CC2650 sensor node.

The Bluetooth SN transmits the data of three sensors to an iPhone7 with iOS 12.0.1 (Apple, Cupertino, CA, USA) using its SensorTag app. Screen shots of the results over time are shown in Figure 15. With a duty cycle of two seconds, and in spite of a high initial peak of current consumption that sets the voltage lower than the “100% battery charge“ measured by the SN, it finally settles in about 76% charge, and maintains this level over time.

### 6.3. Efficiency

The performance of the energy transfer is expressed in terms of the relative efficiency η, defined as the ratio of output and input powers $\eta =P_{o}/P_{in}$. $P_{in}$ is the maximum raw power that the harvester is able to deliver: 0.98 mW (Section 3.2). $P_{o}$ was estimated in each functional stage of the PMIC. During the cold-start and switching intervals, $P_{o}$ is the power injected in $C_{storage}$, and it was obtained by measuring the voltage across it and numerically estimating its through-current $i_{C}$ using the basic model of a super-capacitor (in series with its Equivalent Series Resistance—ESR), by numerically solving the differential equation:

(6) $$v_{bus}=ESR\times i_{C}+\left(1/C_{storage}\right)\int_{0}^{t}i_{C}dt.$$

This estimation is necessary due to the challenge of measuring extremely low currents, something non-viable in this study. $P_{o}$ can then be estimated over the measurement interval of $v_{bus}$, $\left[t_{o},t_{f}\right]$ as:

(7) $$P_{o}\;=\;\frac{1}{t_{f}-t_{o}}\int_{t_{o}}^{t_{f}}i_{C}v_{bus}dt.$$

During cold-start, the efficiency was found to be η = 236.17 μW/980 μW = 0.24. During switching, it was η = 378.36 μW/980 μW = 0.39. For the operational interval, the minimum operational duty cycle indicates the power that the load is absorbing, and is inferred from the current profiles/tables in Section 4. For the minimum duty cycles of 30 s/2 s for the LoRa/Bluetooth boards, the efficiency is estimated as η = 281.6 μW/980 μW = 0.29 and η = 282.1 μW/980 μW = 0.29, respectively. It can be seen that the overall efficiency of the system fluctuates around 30%. This is in agreement with other energy conversion techniques for EH [32]. The results in this section serve to validate the operation of the proposed EH system.

It is to be noted that the relative efficiency estimation performed in this section refers to the output power that can be obtained compared against the total, raw input power that the harvester is able to deliver to a resistive load. This gives an idea of the total energy available from the harvester, in comparison to what is possible to use in practice via the conversion system.

A more absolute assessment of the efficiency, i.e., the actual ratio between the output and input power would require the measurement of the input current, which is more challenging to obtain by standard methods. The introduction of any transducer in the input of the system causes a loading effect that has been experimentally shown to alter the behaviour of the overall system. This is due to consumption by this impedance of a non-trivial amount of the (already low) energy available; moreover, it adds unacceptable uncertainty to the variable under measurement. Special methods are needed to deal with this type of measurement, which were not accessible by the authors during this study.

Finally, an approximation of the power consumed only by the control circuitry in Figure 9 can be obtained by multiplying the voltage from which the control logic feeds ($v_{bus}$), times the current consumption of the PMIC during its operating mode at open load ([28]). In this application, this is approximately: 3.7 V × 2.6 μA = 9.62 μW.

## 7. Conclusions

This paper reported the successful operation of two IoT sensor nodes, which are continuously powered by real industrial vibrations from a typical air compressor. The system makes use of a multi-modal EM-VEH device. A customised power conditioning system for this harvester was introduced. The system is able to power a LoRaWAN sensor node with a duty cycle of 30 s, which is far more frequent than the 15 min usually expected in the protocol. Likewise, a Bluetooth node was energised with a minimum duty cycle of 2 s. The efficiency of the overall energy transfer is about 30%, which is up to the standard in this particular field of energy harvesting. The significance of providing sufficient energy to receive and transmit data at intervals of less than one minute, with wireless sensors of different communication protocols powered by this EM-VEH technique, is the demonstration that standard industrial vibrations can serve as a reliable source of energy to power common wireless sensor nodes for the IoT, without the need of batteries, and independently of the wireless technology used. In particular, the proposed combination of the EM-VEH device and power management system can be used as a design reference that helps to reduce the time to market in industrial applications using sensors in environments at the edge of the IoT.

## Figures and Tables

**Table 1:** Average LoRa board power consumption.

**Sleep Time**	10 s	40 s	2 min	5 min	15 min
**Power (μW)**	714.5	205.9	71.0	28.7	9.6

**Table 2:** Average bluetooth board power consumption.

**Sleep Time (s)**	0.5	1	1.5	2	2.5
**Power (μW)**	693.4	419.6	328.3	282.7	255.3
